# The RNA ac^4^C writer NAT10-dependent post-transcriptional regulations are prerequisites for zygotic splicing activation in mouse

**DOI:** 10.1038/s41419-026-09186-y

**Published:** 2026-08-17

**Authors:** Wen-Jing Wang, Yu-Ke Wu, Yun-Wen Wu, Hua Zhang, Zhi-Yan Jiang, Rui-Bao Su, Lu Chen, Heng-Yu Fan

**Affiliations:** 1 MOE Key Laboratory for Biosystems Homeostasis and Protection and Innovation Center for Cell Signaling Network, Life Sciences Institute, Zhejiang University, Hangzhou, 310058 China; 2 Zhejiang Key Laboratory of Precise Protection and Promotion of Fertility, Department of Obstetrics and Gynecology, Sir Run Run Shaw Hospital, School of Medicine, Zhejiang University, Hangzhou, 310016 China; 3 Fertility Preservation Laboratory, Reproductive Medicine Center, Guangdong Second Provincial General Hospital, Guangzhou, 510317 China; 4 State Key Laboratory of Female Fertility Promotion, Center for Reproductive Medicine, Department of Obstetrics and Gynecology, Peking University Third Hospital, Beijing, 100191 China

**Keywords:** Embryonic induction, Reprogramming, Epigenetic memory

## Abstract

Programmed establishment of the zygote epitranscriptome orchestrates epitranscriptome fidelity and developmental potency acquisition during mammalian preimplantation embryogenesis. N4-acetylcytidine (ac^4^C) is a conserved RNA modification catalyzed by N-acetyltransferase 10 (NAT10), the currently established mammalian RNA ac^4^C writer, but its maternal function during the maternal-to-zygotic transition remains incompletely understood. In this study, we found that oocyte-specific *Nat10* ablation from primary follicle stage causes female infertility with embryonic developmental arrest predominantly at the 2-cell stage. Notably, the maternal protein NAT10 is essential for ac^4^C deposition on zygotic genome activation (ZGA) transcripts and maternal mRNAs, and the loss of *Nat10* can lead to abnormal accumulation of maternal transcripts and major ZGA inactivation. By interrogating ac^4^C LACE-seq, RNA-seq, and Ribo-lite data, we identify splicing-factor transcripts as prominent NAT10-associated ac^4^C targets whose mRNA abundance and ribosome occupancy are reduced after maternal *Nat10* deletion. These molecular changes are accompanied by impaired nuclear speckle organization and widespread disruption of detectable alternative-splicing events during zygotic splicing activation. Together, our findings demonstrate that the RNA ac^4^C writer NAT10 is essential for the post-transcriptional regulation of mouse zygotic splicing activation and provide valuable view for further exploration of the epigenetic mechanism during maternal-to-zygotic transition.

**Figure:**
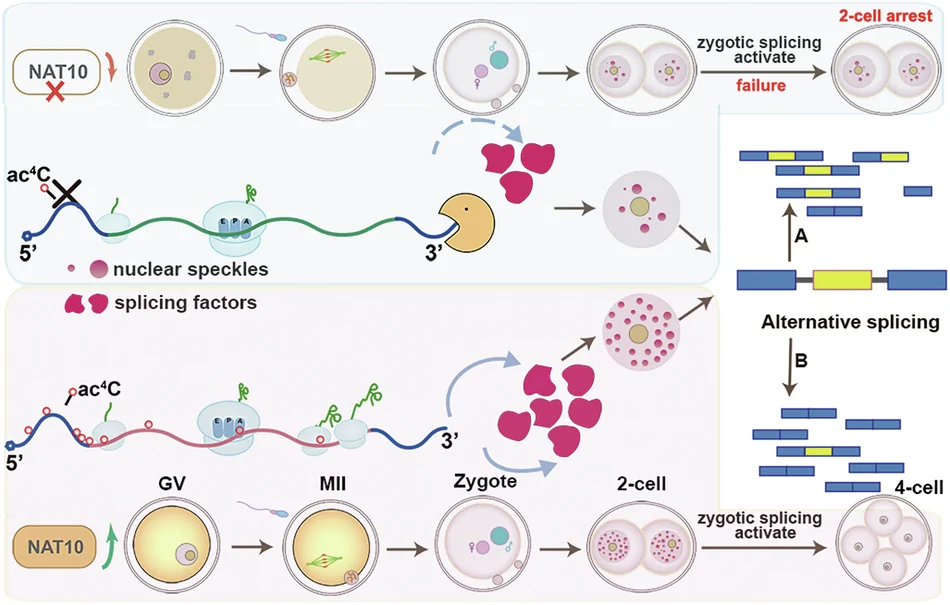
**Schematic illustration showing the function of maternal NAT10-mediated mRNA ac**^**4**^**C in mouse zygotes for post-transcriptional regulation during maternal-to-zygotic transition and zygotic splicing activation**. In summary, NAT10-mediated ac^4^C modification is essential for correctly post transcriptional regulation of zygotic splicing factors’ mRNA stability and translation efficiency during mammalian preimplantation development, throughout this process, alternative splicing is dynamically regulated to generate distinct protein isoforms. The production of the splice variant mediates critical developmental pathways, thereby orchestrating normal embryonic morphogenesis. Upon maternal NAT10 knockout, maintenance of ac^4^C modifications on key transcripts is compromised, which destabilizes their expression. This perturbation triggers defective nuclear speckle assembly and consequently misdirects alternative splicing programs, which ultimately leads to embryogenesis arrests at two-cell block.

**Schematic illustration showing the function of maternal NAT10-mediated mRNA ac**^**4**^**C in mouse zygotes for post-transcriptional regulation during maternal-to-zygotic transition and zygotic splicing activation**. In summary, NAT10-mediated ac^4^C modification is essential for correctly post transcriptional regulation of zygotic splicing factors’ mRNA stability and translation efficiency during mammalian preimplantation development, throughout this process, alternative splicing is dynamically regulated to generate distinct protein isoforms. The production of the splice variant mediates critical developmental pathways, thereby orchestrating normal embryonic morphogenesis. Upon maternal NAT10 knockout, maintenance of ac^4^C modifications on key transcripts is compromised, which destabilizes their expression. This perturbation triggers defective nuclear speckle assembly and consequently misdirects alternative splicing programs, which ultimately leads to embryogenesis arrests at two-cell block.

## Introduction

The epitranscriptome encompasses various dynamic and reversible chemical modifications of RNA transcripts that play essential roles in regulating gene expression and cell fate. More than 170 types of RNA modifications have been discovered, several of which are present in mRNA and regulate gene expression [1], including N^6^ -methyladenosine (m^6^A), pseudouridine (ψ), N^1^-methyladenosine (m^1^A), 5-methylcytidine (m^5^C), N^4^-acetylcytidine (ac^4^C), N7-methylguanosine (m^7^G), and ribose methylations (N_m_), etc. These modifications are essential for mRNA transcription, nuclear export, translation, and degradation, and affect various cellular and biological processes [2–4]. Among these RNA modifications, m^6^A is the most widespread internal mRNA modification and is involved in many critical aspects of RNA metabolism that have been investigated for a broad range of critical functions, including early development, viral infections, and cancer [4–8]. The ac^4^C modification is the third most abundant chemical modification after m^6^A and ψ [9, 10]. It represents the only identified RNA acetylation event detected to date in eukaryotes. In contrast to m^6^A, the pathways and mechanisms that regulate the presence of ac^4^C in specific transcripts, as well as its functions in various physiological and disease contexts, continue to be explored [10–12]. With the advancement of high-throughput sequencing technologies in recent years, the study of epitranscriptomic modifications has accelerated significantly. Among them, ac^4^C has attracted significant research interest. Further study reported that ac^4^C was the first mRNA acetylation event to be studied. The ac^4^C modification is catalyzed by a single enzyme, N-acetyltransferase 10 (NAT10), and the most ac^4^C peaks are enriched in the 5′ untranslated regions (5′ UTR) and coding sequences (CDSs) throughout the length of mRNAs, which within CDS promotes translation and mRNA stability [12, 13].

Fertilization initiates maternal-to-zygotic transition (MZT) within 2–3 days post-fertilization in mice. It is a fundamental process in which transcriptionally dormant oocytes undergo a remarkable shift from a highly specialized, non-proliferative, meiotic state to totipotent, mitotic embryos [14–17]. Post-transcriptional regulation is critical for managing maternal mRNA storage, decay, and sequential activation of both maternal and zygotic transcripts during this transition [18–20]. In early preimplantation mammalian embryos, while DNA and chromatin undergo profound reorganization with highly asymmetric epigenetic modifications, the mechanisms controlling RNA modification remain elusive [21, 22]. These epigenetic changes, which are involved in establishing stable heritable markers, are intrinsic to development and cell differentiation [9, 23]. Recent studies have reported that similar to m^6^A, dynamic ac^4^C modification regulates various aspects of RNA metabolism, such as mRNA stability and translation, and impacts diverse biological and pathological processes, including cancer, inflammation, immune regulation, neurological disorders, osteogenic differentiation and viral infection [3, 24–30]. For instance, a few studies have underscored the significant involvement of ac^4^C modification and NAT10 in mammalian development. NAT10 is an ac^4^C writer that is required for folliculogenesis [31], oocyte maturation, and maintenance of female fertility [24, 32, 33]. In male germ cells NAT10 is particularly abundant in the testes where *Nat10* ablation leads to spermatogenesis defects and affects male fertility [34]. In addition, NAT10 modulates embryonic stem cell pluripotency [27].

During the early stages of mammalian embryogenesis, which rely primarily on rapidly degrading maternal mRNAs, RNA epigenetic regulation, particularly chemical modification regulation, critically dictates the functionality of maternally inherited transcripts [2]. The essential functions of m6A during the maternal-to-zygotic transition have been well established, including roles in maintaining maternal mRNA stability in oocytes and promoting timely RNA decay after fertilization [19]. Thus, the molecular mechanism of by which ac^4^C regulates RNA metabolism in oocytes and early embryos, zygotic genome activation (ZGA), and preimplantation remains largely ambiguous and requires further investigation.

Transcriptomic profiling of murine and human early embryos has revealed dynamic alternative splicing during MZT, revealing zygotic splicing activation (ZSA) as a previously uncharacterized post-transcriptional process following embryonic genome activation [35, 36]. This mechanism entails maternal-to-zygotic replacement of splicing factors and is physiologically indispensable for embryogenesis [37]. In addition to transcriptional control, precise isoform expression mediated by alternative splicing (AS) is required for preimplantation development. In higher eukaryotes, AS diversifies the proteome through multi-isoform mRNA generation from single genes, frequently altering the predicted domain architecture of the resulting proteins [38]. Nevertheless, the regulatory determinants governing alternative splicing and genomic integrity processes during the MZT remain incompletely characterized. A recent report has shown that NAT10 phase separation regulates YTHDF1 splicing to promote gastric cancer progression, suggesting the potential involvement of NAT10-mediated ac^4^C in alternative splicing regulation during ZSA [39].

Therefore, we wondered whether NAT10-mediated accumulation of RNA acetylation marks in oocytes is necessary for the coordination of alternative splicing with early embryonic development. In this study, we report that *Nat10*-knockout during oocyte growth results in female infertility with developmental arrest at the 2-cell stage. By integrating ac^4^C LACE-seq, RNA-seq, low-input ribosome profiling and functional assays, we investigated how maternal NAT10 contributes to post-transcriptional regulation during the maternal-to-zygotic transition.

## Material and methods

### Animals

The *Nat10*^*fl/fl*^ mouse strain was generated as previously described [34]. All the mouse strains had a C57BL/6 J genetic background. Founder mice were identified by PCR genotyping and crossed with WT C57B6 partners to ensure germline transmission and avoid potential off-target effects. All animal experiments were conducted in accordance with the guidelines and regulations of Zhejiang University, and the experimental protocol (ZJU20250080) was approved by the Zhejiang University Institutional Animal Care and Research Committee. All experimental procedures were performed on female mice. Sample size was established according to previous studies [32]. No criteria for animal exclusion were set. Investigators were not blinded to group allocation or outcome assessment. The primers used for genotyping are listed in Supplementary Table 1.

### Superovulation and fertilization

For superovulation, female mice (24–28 days old) were intraperitoneally injected with 5 IU of pregnant mare’s serum gonadotropin (PMSG, Ningbo Sansheng Pharmaceutical Co., Ltd., China). After 44 h, the mice were injected with 5 IU human chorionic gonadotropin (hCG; Ningbo Sansheng Pharmaceutical). After an additional 16 h, the oocyte-cumulus complex was surgically removed from the oviducts and the number of oocytes was counted after digestion with 0.3% hyaluronidase (Sigma-Aldrich, St Louis, MO, USA). Oocyte morphology was observed and images were acquired using a Nikon SMZ1500 stereoscope.

To obtain early embryos, female mice were mated with 10- to 12-week-old WT males. Successful mating was confirmed by the presence of vaginal plugs. Embryos were harvested from the oviducts at the indicated time points after hCG injection.

### Histological analysis and immunohistochemistry

Ovaries were fixed overnight in 10% phosphate-buffered saline (PBS)-buffered formalin at 4 °C. After dehydration, the ovaries of the mice were embedded in paraffin and sectioned at a thickness of 5 μm. Sections were deparaffinized in xylene three times (5 min each), rehydrated twice in anhydrous ethanol and 95% ethanol (5 min each), and stained with hematoxylin and eosin.

For IHC staining, the sections were incubated in 3% H_2_O_2_ (v/v) for 10 min, cooled down to room temperature after boiling in 10 mM sodium citrate buffer (pH 6.0) for 15 min, washed, blocked with 10% donkey serum for 60 min, and incubated with primary antibodies overnight at 4 °C. The slides were washed and incubated with biotin-labeled secondary antibodies. The antibodies were further detected using the VECTASTAIN® ABC kit and 3,3′-diaminobenzidine peroxidase substrate kit (Vector Laboratories, Burlingame, CA, USA). The slides were counterstained with hematoxylin and eosin, and imaged using a Nikon 80i microscope equipped with a camera.

### Confocal microscopy for mouse oocytes and embryos

Oocytes and embryos were fixed in PBS-buffered 4% paraformaldehyde (PFA) for 30 min at room temperature, followed by permeabilization with 0.2% Triton X-100. After being blocked with 1% BSA in PBS, oocytes were incubated with primary antibodies diluted in blocking solution at room temperature for 1 h or overnight at 4 °C. After being washed three times with PBS, oocytes were labeled with secondary antibodies for 45 min at room temperature, and then counterstained with 5 μg/ml of 4′,6-diamidino-2-phenylindole (DAPI) or propidium iodide (PI) (Molecular Probes, Life Technologies) for 10 min. The oocytes were mounted on glass slides with SlowFade Gold Antifade Reagent (Life Technologies) and examined under a confocal laser scanning microscope (Zeiss LSM 710, Carl Zeiss).

### Preparation of mRNAs for microinjections

The expression vectors were linearized using appropriate restriction enzymes and 5′-capped mRNAs were in vitro transcribed using the T7 mMessage mMACHINE Kit (Invitrogen, Carlsbad, CA, USA; AM1340). Poly(A) tails (~200–250 bp) were added using an mMACHINE kit (Invitrogen, AM1350). The in vitro–transcribed mRNAs were resuspended in nuclease-free water using the lithium chloride precipitation method.

### Microinjection of zygotes

For microinjection, mouse zygotes were obtained by superovulation of 3–4-week-old females mated with males of the same strain. For *Nat10* overexpression, 800 ng/μL mRNA was injected into the cytoplasm of zygotes with well-recognized pronuclei, in M2 medium (Sigma). Injected zygotes were transferred into KSOM medium for further culture at 37 °C with 5% CO_2_.

### Western blotting

Ovaries or oocytes were prepared by lysing and denaturing in 2× sodium dodecyl sulfate (SDS) sample buffer (containing β-mercaptoethanol) and heated for 5 min at 95 °C. Proteins were separated by SDS-polyacrylamide gel electrophoresis and electrophoretically blotted onto microporous polyvinylidene fluoride (PVDF) membranes (Millipore Corp., Bedford, MA, USA) under a constant current, followed by blocking in TBST containing 5% skim milk (BD, Franklin Lakes, NJ, USA) for 30 min. After probing with primary antibodies, the membranes were washed with TBST and incubated with horseradish peroxidase-conjugated secondary antibodies (Jackson ImmunoResearch Laboratories, West Grove, PA, USA) for 1 h, followed by three washes with TBST. Bound antibodies were detected using an enhanced chemiluminescence western blotting substrate (Thermo Fisher Scientific, Waltham, MA, USA; 32106) or SuperSignal West Femto maximum sensitivity substrate (Thermo Fisher Scientific Inc.). The primary antibodies and dilutions used are listed in Supplementary Table 2.

### Immunoprecipitation (IP)

The ovaries or oocytes were lysed in lysis buffer (50 mM Tris-HCl [pH 7.5], 150 mM NaCl, 10% glycerol, and 0.5% NP-40; protease and phosphatase inhibitors were added before use). After centrifugation at 12,000 × *g* for 10 min, the supernatant was subjected to immunoprecipitation on an anti-NAT10, anti-SRSF2 or control IgG antibody. After incubation at 4 °C for 4 h, the Protein A/G beads were washed three times with lysis buffer. SDS sample buffer was added to the beads and the eluates were used for western blot analysis.

### Detection of protein synthesis in embryos

Embryos were cultured in M16 medium supplemented with 50 μM L- homopropargylglycine (HPG) for 1 h. HPG was detected using Click-iT® HPG Alexa Fluor® Protein Synthesis Assay Kit (Life Technologies), according to the manufacturer’s instructions. The mean cytoplasmic signal was measured across the middle of each oocyte/embryo and quantified using the ImageJ software.

### RNA isolation and RT-qPCR

Oocytes (10 oocytes per sample) were directly lysed with 4.2 μL of lysis buffer (containing 0.2% Triton™ X-100, RNase inhibitor, dNTPs, and oligo-dT primers). Total RNA from oocytes was reverse transcribed using the PrimeScript II 1st strand cDNA synthesis kit (Takara, Kusatsu, Japan; 6210 A). Random primers (Takara, 3801) were used to guide reverse transcription. qPCR was performed using Power SYBR® Green PCR Master Mix (Thermo Fisher Scientific, 4367659) and an Applied Biosystems 7500 Real-Time PCR System. Relative transcription levels were normalized to endogenous *Gapdh* and actin levels, followed by subsequent determination of fold change. Each RT-qPCR experiment was performed with at least three independent replicates. All primers used are listed in Supplementary table 1.

### Ribo-lite library preparation and sequencing

Ribo-lite was adapted from conventional Ribo-seq [40]. Samples were lysed in 310 μL ice-cold lysis buffer (20 mM Tris-HCl pH 7.4, 50 mM NaCl, 5 mM MgCl_2_, 1 mM DTT, 100 μg/ml CHX, 1% Triton; 25 U/mL Turbo DNase (Ambion, AM2239)) for 10 min on ice. The lysate was clarified for 10 min at 20,000 × *g* at 4 °C, then treated with 1 μL RNase I 100 U/mL (Ambion, AM2295) and incubated at room temperature for 45 min with gentle mixing. For 107 cells, 7 μL RNase I was added. The digested extracts were stopped by the addition of 10 μL SUPERase•In (Ambion, AM2696) and were overlaid onto a 700 μL sucrose cushion of 1 M sucrose in 20 mM Tris–HCl pH 7.4, 150 mM NaCl, 5 mM MgCl_2_, 1 mM DTT, 100 μg/mL CHX and 20 U/mL SUPERase•In (Ambion, AM2696) for 4 h at 260,000 × *g*, 4 °C in MLA-150 rotor in Beckman Optima MAX-XP ultracentrifuge. The supernatant was removed, and the pellet was resuspended in 50 μL pellet buffer (10 mM Tris [pH 7.5] and 1% SDS). RPFs were extracted from pellet buffer by using 1 ml TRIzol (life tech, 15596018) and 200 μL chloroform. The aqueous phase was precipitated by adding 750 μL isopropanol and 20 μg glycogen. Precipitation was carried out at −20 °C overnight, and RPFs were then pelleted by centrifugation for 40 min at 12,000 × *g*, 4 °C. RPF was resuspended in 6 μL nuclease-free water and mixed with 6 μL Gel Loading Buffer II (Thermo Fisher, AM8546G). Library structuring was performed as described previously. The count list of Ribo-seq is listed in Supplementary Table 3.

### RNA-seq

RNA was extracted as previously described for acetylated RNA immunoprecipitation, and three replicates were prepared for each sample. RNA-seq was performed using the Smart-seq2 method, with minor modifications. Briefly, RNAs were incubated with polyT primers in a lysis buffer to capture polyadenylated mRNAs. After 3 min of incubation at 72 °C, the Smart-seq2 reverse transcription reactions were performed. After the first-strand reaction, cDNA was amplified using a limited number of cycles (approximately 13 cycles). Sequencing libraries were constructed from 1 ng of amplified cDNA using the TruePrep DNA Library Prep Kit V2 for Illumina (Vazyme, TD503) according to the manufacturer’s instructions. Barcoded libraries were pooled and sequenced on the Illumina HiSeq X Ten platform in the 150 bp paired-end mode.

### RNA-seq analysis

Raw reads were trimmed with Trim-Galore v0.6.7, and mapped to the mm10 genome using STAR v2.7.10a. Uniquely mapped reads were used to quantify gene expression using FeatureCounts v2.0.2, and were further normalized to ERCC spike-in. The ERCC table was constructed as described above using the ERCC reference genome, and the percentage of ERCC reads was used for data calibration. The expression level of each transcript was quantified to the normalized FPKM (listed in Supplementary Tables 4). Differential gene expression analysis was performed using the DESeq2 R package, and an adjusted *P* value of <0.05, and an absolute Log2(fold change of cKO/WT) > 1 were used as statistical significance to identify differentially expressed genes (listed in Supplementary Tables 5).

### ac^4^C LACE-seq

For ac^4^C LACE‑seq [32], approximately 50 oocytes were collected into 1.5‑ml Eppendorf LoBind tubes and briefly pelleted by centrifugation. Cells were lysed on ice for 10 min in 50 μl of wash buffer, followed by the addition of 1 μl RNase inhibitor (Ambion, AM2696) and 4 μl RQ1 DNase (Promega, M6101), with incubation at 37 °C for 3 min. The reaction was immediately placed on ice for 3 min to halt enzymatic activity. Subsequently, 2 μg of ac^4^C antibody was introduced, and the mixture was rotated at 4 °C for 1 h to allow immunoprecipitation. Cross‑linking was achieved by exposing the samples to UV‑C irradiation (400 mJ) twice on ice. Protein A/G magnetic beads (10 μl) were then added, followed by a 2‑h rotation at 4 °C. Following stringent washes, the immunoprecipitated RNA was fragmented, subjected to 3′‑dephosphorylation, and ligated to appropriate linkers. Reverse transcription was performed directly on the bead surface. First‑strand cDNA was recovered and further captured using streptavidin C1 beads for pre‑PCR amplification. A 3′‑cDNA linker was subsequently appended to generate double‑stranded DNA suitable for in vitro transcription. Transcription products were treated with Turbo DNase to remove templates and purified using Agencourt RNAClean beads per the manufacturer’s protocol. The resulting linear amplified RNA was reverse‑transcribed into cDNA and PCR‑amplified with P7 and barcoded P5 index primers. Finally, amplicons ranging from 250 to 500 bp were gel‑excised from a 2% agarose gel and purified using a Qiagen Gel Extraction Kit (catalog no. 28604).

### ac^4^C LACE-seq peak and motif identification

We used MACS (v.2.2.7.1) software to identify peaks in WT and *Nat10* knockout GV oocytes. The parameters were as follows: --keep-dup all --fe-cutoff 2 -p 0.001 --extrasize 100, and nomodel. Only peaks that appeared in both replicates were retained. For motif analysis, LACE-seq peaks were first extended 30 nt to 5′ upstream, and obtained its corresponding sequence on the mm10 reference genome using bedtools (v.2.30.0) with the following parameters: -fi -bed -fo. Then, MEME (v.4.11.2) software was used to obtain their motif logo using default parameters.

### Analysis of various alternative splicing events

Using replicate multivariate analysis of transcript splicing (rMATS) [41, 42], five different ASEs were detected: SE, A5SS, A3SS, mutually exclusive exons (MXE), and intron retention. Various alternative splicing events were considered when the false-discovery rate (FDR) was <0.05 and the IncLevelDifference was >0.1 or <−0.1.

### Quantification and statistical analysis

Each experiment was repeated at least three times. All data represent at least three independent experiments. No samples or animals were excluded from the analyses in this study. All experimental subjects met the preestablished health and viability criteria throughout the experimental period. Results are given as mean ± SEM. Student’s *t* test and Mann‒Whitney Wilcoxon test were used to compare continuous variables between the two groups. Analysis of variance (ANOVA) was used to estimate the variance among multiple groups. Fisher’s exact test was used to compare categorical variables. P-values in multiple tests were adjusted to the false discovery rate (FDR) using the Benjamini-Hochberg method. All statistical analyses were performed using R software (version 4.0.3). Values of *p* < 0.05 were considered to be statistically significant. In the graph, * represents *p* < 0.05, ** represents *p* < 0.01, *** represents *p* < 0.001, **** represents *p* < 0.0001.

## Results

### The dynamic expression of *Nat10* in mammalian preimplantation embryos along with changes in ac^4^C abundance

**Fig. 1:**
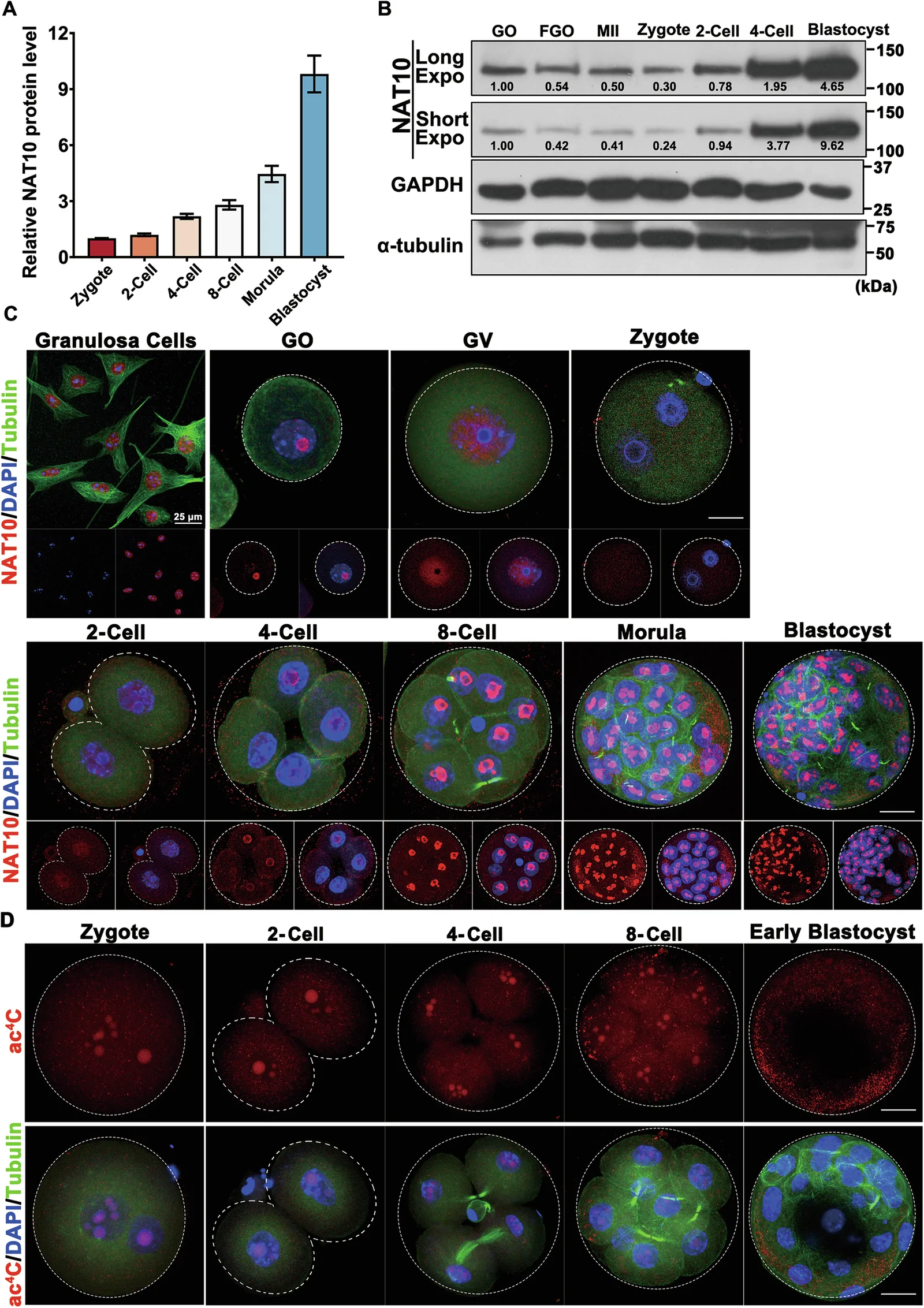
Dynamics of NAT10 and ac^4^C level in mammalian preimplantation embryos. **A** Relative protein expression levels of NAT10 during mouse developmental stages of preimplantation embryos. Data were extracted from published proteome results. The relative expression of NAT10 at the zygote stage was normalized to 1. **B** Western blotting results showing the expression level of NAT10 in mouse oocytes and preimplantation embryos. Each line was loaded with 250 oocytes, and GAPDH and α-tubulin were used as loading controls. **C** Immunofluorescent staining for NAT10 (red) and tubulin (green) in mouse preimplantation embryos. DNA was counterstained with 4’,6-diamidino-2-phenylindole (DAPI, blue). **D** Confocal microscopy images of ac^4^C (red) and tubulin (green) immunofluorescence in mouse preimplantation embryos. DNA was counterstained with DAPI (blue).

To explore the role of ac^4^C in the MZT, we first examined whether and how the only known ac^4^C writer, NAT10, was expressed in mammalian oocytes and early embryos. Existing RNA sequencing (RNA-seq) and proteomic results showed that the expression level of *Nat10* was higher in human early embryos, especially in zygote, 2-cell, and 4-cell embryos, than in oocytes (Fig. S1D), and NAT10 protein level gradually increased in preimplantation embryos (Fig. 1A). Western blotting results indicated that NAT10 protein levels were gradually decreased during the oocyte maturation and maternal-to-zygotic transition (MZT) process. Then NAT10 increased again during early embryonic development and abundantly present in blastocyst (Fig. 1B, Fig. S1A), which is consistent with the findings of previous reports [32, 33, 43]. Immunofluorescence staining analysis revealed that NAT10 was expressed in mouse oocytes and early embryos. However, the subcellular distribution patterns of NAT10 differed slightly at different stages (Fig. 1C). Consistent with previous reports, NAT10 localized to the nucleolus in granulosa cells, growing oocytes, and embryos beyond the 4-cell stage. In contrast, NAT10 was mainly nucleoplasmic in GV oocytes, zygotes, and 2-cell embryos, suggesting a potential role for maternal NAT10 during MZT (Fig. 1C, Fig. S1B). Furthermore, ac⁴C immunofluorescence revealed signals in both the nucleus and cytoplasm of early embryos, with the strongest enrichment in the nucleolus (Fig. 1D, Fig. S1C). These results indicate dynamic changes in NAT10 expression and localization accompanied by altered ac⁴C distribution, suggesting an important role for NAT10 in regulating RNA ac⁴C modification during early embryonic development.

### Maternal *Nat10* deletion does not grossly impair ovulation or in vivo meiotic maturation

**Fig. 2:**
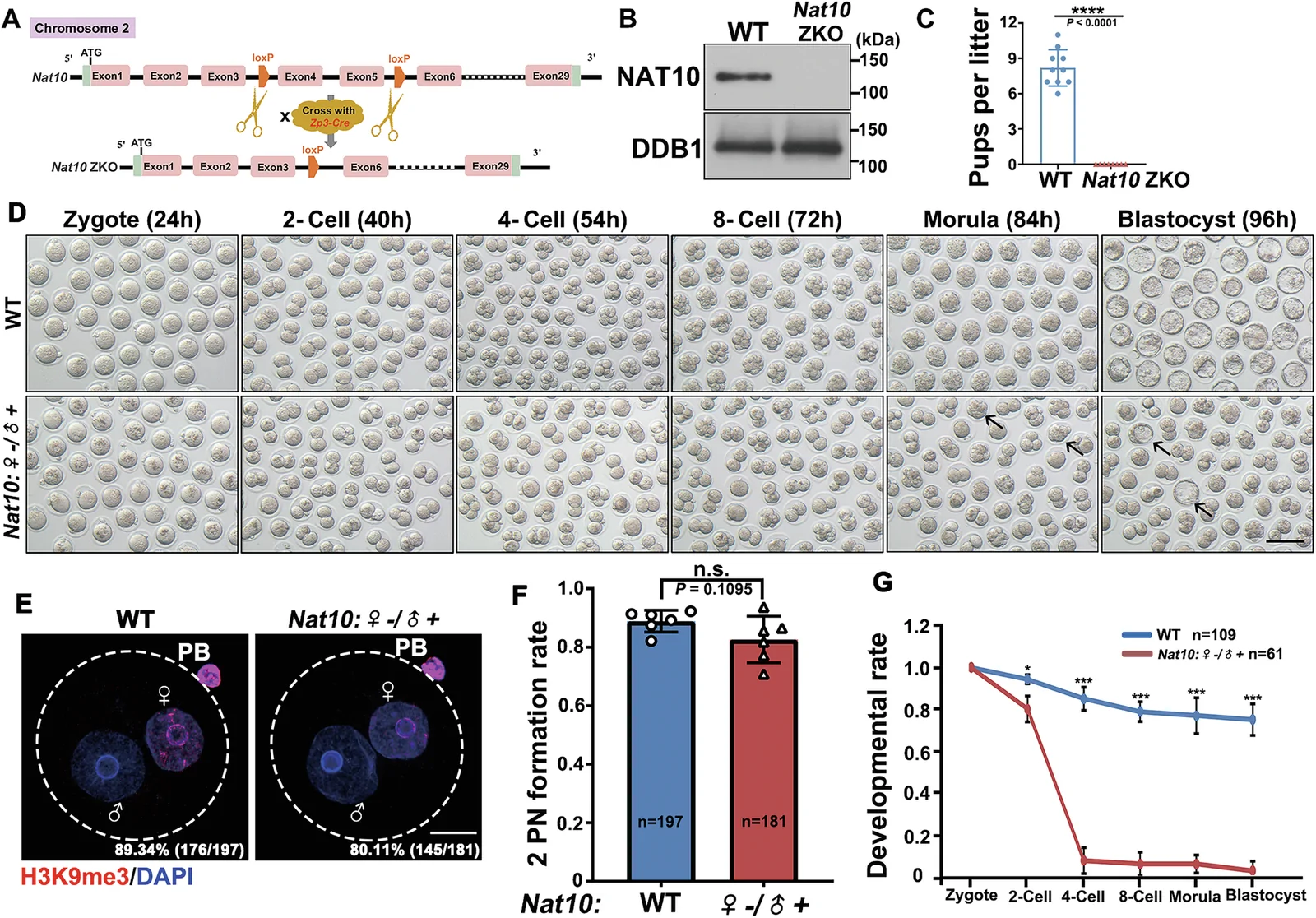
Maternal deletion of *Nat10* arrests early embryonic development beyond the two-cell stage. **A** Schematic of the *Nat10* conditional targeting construct strategy. **B** Western blotting results of NAT10 expression levels in oocytes of WT and *Nat10*^*fl/fl*^*;Zp3-Cre* females showing successful deletion of NAT10 in the primary follicles. DDB1 was used as the loading control. **C** Fertility test analysis showing the average number of pups per litter of the indicated genotypes. WT (*n* = 8) and *Nat10*^*fl/fl*^*;Zp3-Cre* (*n* = 8) female mice were mated with WT males for 5 months, and the total number of pups per mouse was assessed. **D** Morphological images of embryos obtained by in vivo fertilization of mated WT and *Nat10*^*fl/fl*^*;Zp3-Cre* females at the indicated time points after hCG administration and in vitro culture. Scale bar = 100 μm. **E** Immunofluorescence staining results for trimethylated histone H3 at Lys-9 (H3K9me3, red) in WT and *Nat10*^*fl/fl*^*;Zp3-Cre* zygotes 24 h after hCG injection. **F** Rates of pronucleus formation in WT and *Nat10*^*fl/fl*^*;Zp3-Cre* zygotes mated with male WT mice. The number of embryos analyzed is shown (n). ns non-significant according to two-tailed Student’s t-test. Error bars indicate the SEM images. **G** Developmental ratios of preimplantation embryos derived from WT and *Nat10*^*fl/fl*^*;Zp3-Cre* females when WT embryos reached their corresponding stage. The number of embryos analyzed is shown (n). *P < 0.05, ***P < 0.001, ****P < 0.0001 by two-tailed Student’s *t* test. Error bars indicate ± SEM.

To investigate the physiological function of NAT10, we generated a conditional knockout of the NAT10 mouse strain using *Zp3-Cre* and crossed it with *Nat10-*floxed mice (Fig. 2A). In *Nat10*^*fl/fl*^*;Zp3-Cre* mice, *Nat10* was specifically deleted in oocytes beginning at the primary follicle stage. *Nat10*^*fl/fl*^*;Zp3-Cre* female mice were sterile (Fig. 2C). Western blotting and immunostaining confirmed the absence of the NAT10 protein in oocytes (Fig. 2B and Fig. S1E, F). Moreover, ac^4^C signals in *Nat10*-deleted oocytes were significantly decreased (Fig. S3A, B). *Nat10*^*fl/fl*^*;Zp3-Cre* female mice (4-week-old) displayed normal ovarian morphology and histology with comparable weights (Fig. S2A–C). After pregnant mare serum gonadotropin (PMSG) injection, the ovaries of *Nat10*^*fl/fl*^*;Zp3-Cre* (4-week-old) mice normally responded to gonadotropins compared with those of wild-type (WT) mice, and GV oocytes derived from *Nat10*^*fl/fl*^*;Zp3-Cre* mice almost all underwent the NSN-to-SN transition (Fig. S2A–F). *Nat10*^*fl/fl*^*;Zp3-Cre* females ovulated normally, producing comparable numbers of oocytes and ovary weights to WT mice after hCG injection (Fig. S2D, E, S3F). Ovulated *Nat10*^*oo−/−*^ oocytes extruded polar body-1 (PB1) and displayed normal-shaped spindle (Fig. S2G, H). Moreover, *Nat10-*null GV oocytes underwent normal in vitro maturation and maintained normal levels of key maternal fertility factors, including BTG4, CNOT7, and CPEB1 (Fig. S2I, S3C–G), indicating that *Nat10* deletion from primary follicle oocytes does not markedly affect meiotic maturation.

### NAT10 is essential for mouse embryonic development

To investigate why *Nat10*^*fl/fl*^*;Zp3-Cre* mice were infertile, we studied the developmental potential of zygotes derived from *Nat10*-deleted oocytes. These oocytes were fertilized normally and displayed a normal H3K9me3 pattern (Fig. 2D–F). Most maternal *Nat10*-knockout embryos (*Nat10*^*♀−/♂+*^) underwent the first round of cleavage normally, but arrested at the 2-cell stage, whereas WT (*Nat10*^*♀+/♂+*^) embryos developed normally (Fig. 2D, G). Caudal-type homeobox 2 (CDX2) is a functional marker of TE, while NANOG is a functional marker of ICM. A small subset of normally fertilized zygotes derived from *Nat10*^*fl/fl*^*;Zp3-Cre* (hereafter referred to as ZKO) females reached the blastocyst stage but showed disrupted CDX2 and NANOG distribution, indicating impaired trophectoderm (TE) and inner cell mass (ICM) lineage specification (Fig. S2J, K). This suggests that maternal NAT10 loss compromises blastocyst quality in the few embryos that bypass the 2-cell arrest. These results demonstrate that NAT10 is crucial for embryonic development and MZT.

### Loss of maternal *Nat10* leads to abnormal accumulation of maternal transcripts and dysregulation of ZGA transcripts

**Fig. 3:**
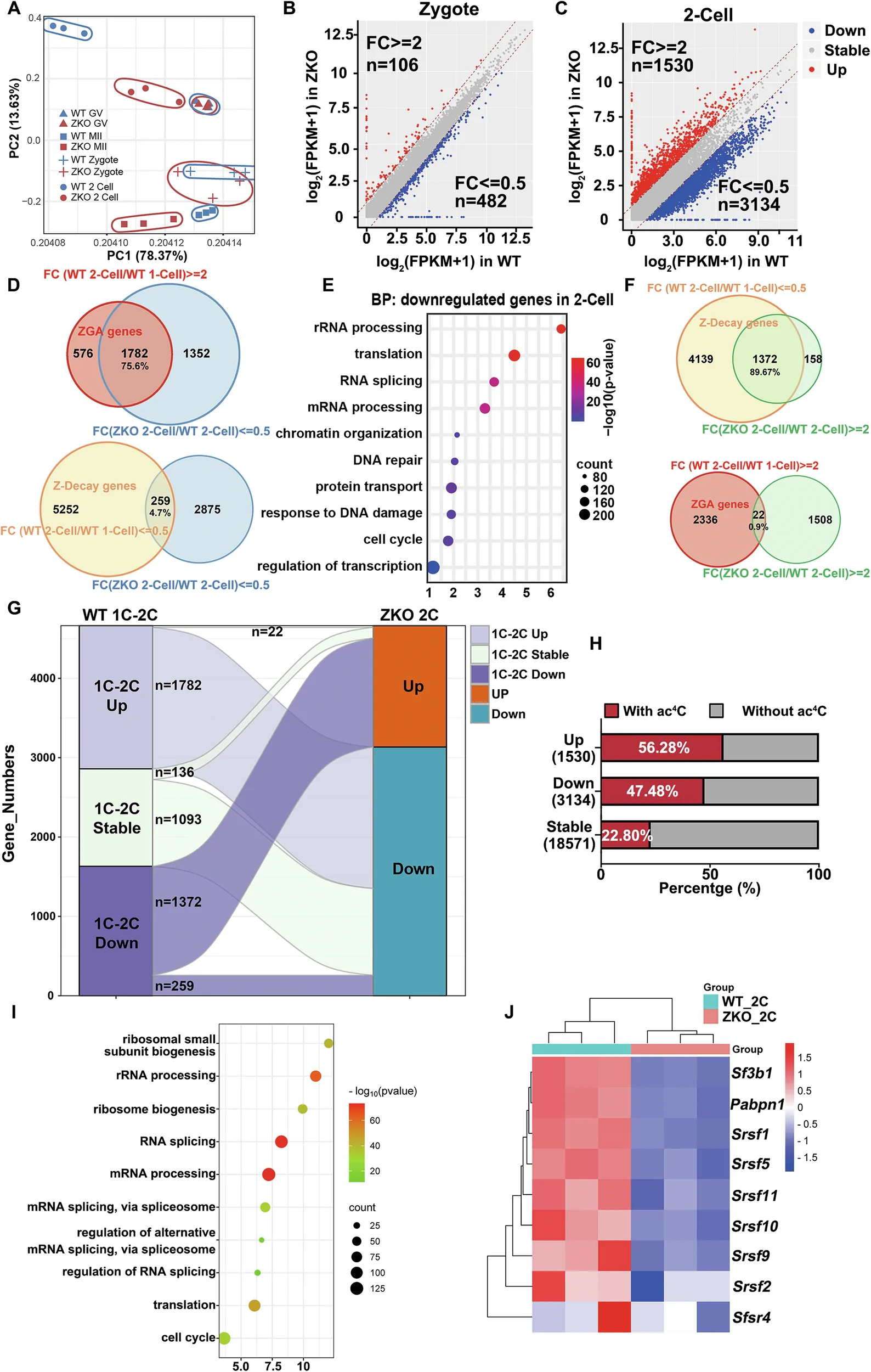
Transcriptomic analyses of maternal *Nat10*-depleted embryos during the MZT. **A** Principal component analysis (PCA) plot visualizing individual transcriptomes by developmental stage and genotype. n = 3 biological replicates per genotype. **B**, **C** Scatter plots comparing gene expression levels of WT and *Nat10*^*fl/fl*^*;Zp3-Cre* embryos at the 1-cell (**B**) and 2-cell (**C**) stages. Three RNA-seq replicates were generated for differential gene expression analysis. **D** Venn diagrams showing the overlap in transcripts that were significantly increased during 1-cell to 2-cell transition (fold change (2-cell/1-cell)>=2) of WT embryos and genes downregulated in this process after *Nat10* maternal knockout. **E** Gene Ontology biological processes enriched for significantly downregulated genes after maternal *Nat10* deletion at 2-cell stage. **F** Venn diagrams showing the overlap of transcripts that were decreased in WT 2-cell and increased in *Nat10*^*♀+/♂+*^ embryos during 1-cell the 2-cell transition. **G** Sankey diagrams showing the expression patterns of transcripts at the 1-cell and 2-cell stages in WT and *Nat10*^*fl/fl*^*;Zp3-Cre* mice. Each rectangle represents a specific gene category. **H** Bar plot showing the percentage of transcripts with ac^4^C modification among the upregulated, downregulated, and stable transcripts in the maternal *Nat10* knockout. **I** Gene Ontology analysis of transcripts with ac^4^C modification and downregulated in 2-cell embryos derived from *Nat10*^*fl/fl*^*;Zp3-Cre* females mated with WT males. **J** Heatmap of selected key genes from (**I**) showing distinct expression characteristics in 2-cell embryos derived from WT and *Nat10*^*fl/fl*^*;Zp3-Cre* females mated with WT males.

To investigate the direct or indirect effects of maternal *Nat10* deletion on the MZT, we performed RNA-seq on GV oocytes, MII oocytes, zygotes, and 2-cell embryos of WT and *Nat10*^*fl/fl*^*;Zp3-Cre* mice. Replicates were highly correlated, and principal component analysis (PCA) showed only minor differences between WT and ZKO samples at the GV, MII, and zygote stages, whereas 2-cell embryos were clearly separated (Fig. 3A, Fig. S4A). Consistently, gene expression was minimally affected before the 2-cell stage but was markedly altered in ZKO 2-cell embryos. A large number of genes were differentially expressed: 3134 transcripts were downregulated, and 1530 transcripts were upregulated in *Nat10*^*♀−/♂*^ 2-cell embryos (Fig. 3B, C and Fig. S4B, C). Using transcriptome data, we identified 2358 ZGA genes that were upregulated after fertilization, and 5511 zygotic decay (Z-Decay) genes that were dramatically downregulated from zygote to 2-cell stage. Comparative analysis showed that most ZGA transcripts were aberrantly downregulated in *Nat10*^*♀−/♂*^ 2-cell embryos, whereas nearly all aberrantly upregulated transcripts overlapped with Z-decay genes (Fig. 3D, F). In contrast, downregulated transcripts showed little overlap with Z-decay genes, and upregulated transcripts showed low similarity to ZGA genes. These dysregulated transcripts did not markedly affect normal M-decay progression, and Z-decay transcripts were normally expressed during meiotic maturation (Fig. S4D–F). The alluvial diagram shows the dynamics of differentially expressed transcripts from the zygotes to 2-cell embryos stages (Fig. 3G). Gene ontology (GO) analysis of the downregulated genes in *Nat10*^*♀−/♂*^ 2-cell revealed that clustering occurred during rRNA processing, translation, and RNA splicing (Fig. 3E).

In addition, we investigated whether the enrichment of ac^4^C was different between dysregulated and stably expressed transcripts. The percentages of ac^4^C^+^ transcripts in the upregulated and downregulated classes were significantly higher than those in the stable class (Fig. 3H). GO analysis of the 1,488 (47.48%) downregulated transcripts with ac^4^C modification showed that the genes were enriched in rRNA processing, RNA splicing, ribosome biogenesis, and cell cycle (Fig. 3I). Moreover, the expression levels of some key genes involved in RNA splicing and mRNA processing were significantly reduced in the *Nat10*^*♀−/♂*^ 2-cell compared with WT (Fig. 3J).

### Maternal NAT10 is required for proper nuclear speckle organization in 2-cell embryos

**Fig. 4:**
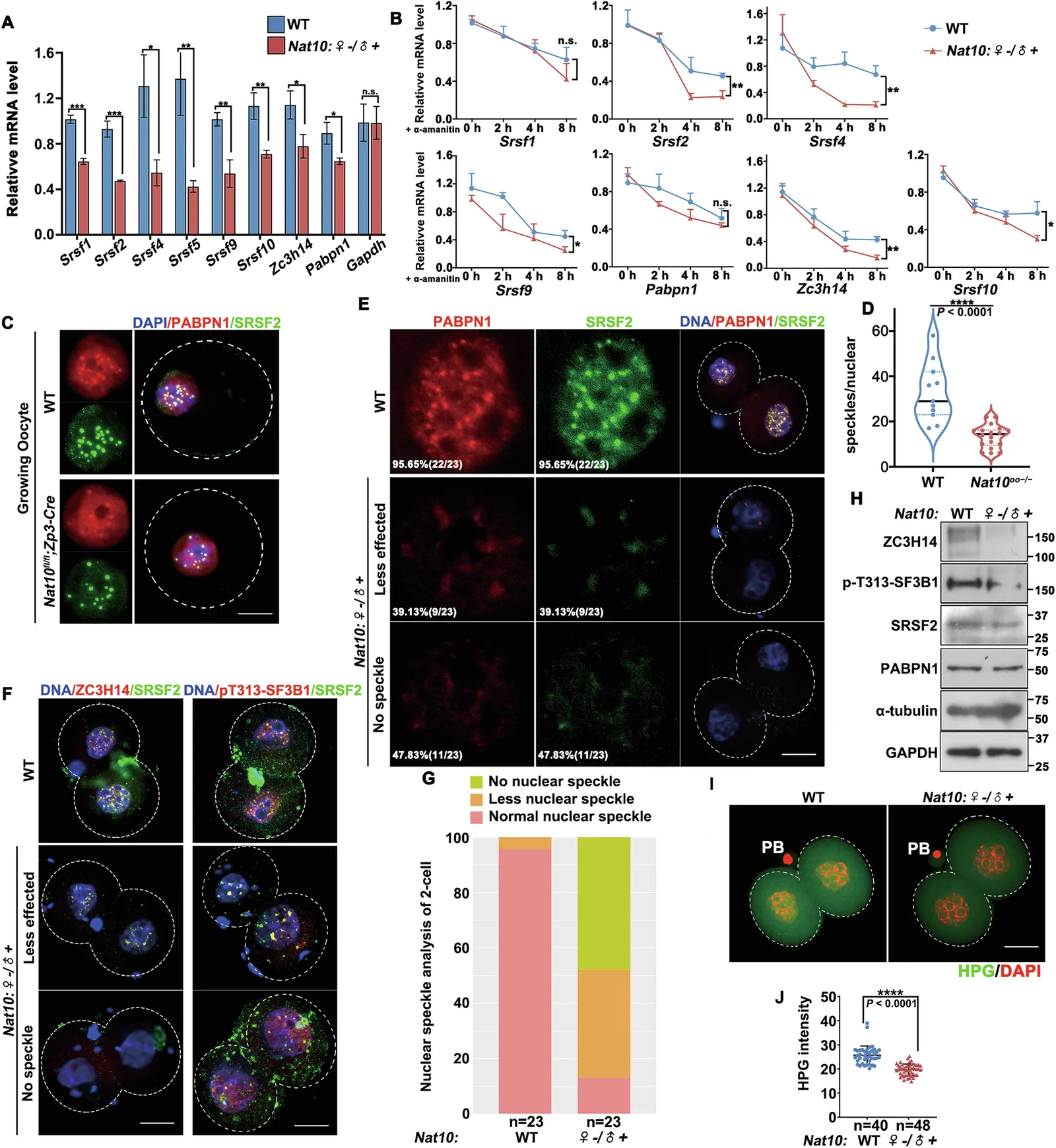
Impact of maternal *Nat10* deletion on nuclear speckles expression. **A** Quantitative RT-PCR results of the relative expression levels of selected functional genes in 2-cell embryos derived from WT and *Nat10*^*fl/fl*^*;Zp3-Cre* females mated with WT males. **B** Quantitative RT-PCR results showing the effect of maternal *Nat10* knockout on the stability of these functional transcripts in 2-cell embryos of WT and *Nat10*^*fl/fl*^*;Zp3-Cre* under α-amanitin treatment. **C** Double immunofluorescence staining of PABPN1 and nuclear speckle marker SRSF2 in growing oocytes derived from WT and *Nat10*^*fl/fl*^*;Zp3-Cre* mice. Scale bar, 20 μm. **D** Quantitative foci analysis of nuclear speckle marker SRSF2. **E**, **F** Immunofluorescence results showing the speckle distribution of PABPN1 (**E**), p-T313-SF3B1, ZC3H14 (**F**), and SRSF2 in 2-cell embryos derived from WT and *Nat10*^*fl/fl*^*;Zp3-Cre* females mated with WT males. Scale bar, 20 μm. The number of analyzed 2-cell embryos is indicated (n). **G** Quantitative nuclear speckle analysis of nuclear speckle marker SRSF2 in (**E**, **F**). 2-cell embryos collected from WT and *Nat10*^*fl/fl*^*;Zp3-Cre* females were mated with WT male mice in three independent experiments. The 2-cell embryos number is 23. **H** Western blot showing the protein expression levels of ZC3H14, p-T313-SF3B1, PABPN1, and SRSF2 in 200 WT and maternal NAT10-deleted 2-cell embryos. α-tubulin and GAPDH were blotted as loading control. **I** HPG staining results showing protein synthesis activity at 2-cell stage in WT and maternal NAT10-deleted 2-cell embryos. Scale bar, 20 μm. **J** Quantification of fluorescence intensity (**I**). ns not significant; *P < 0.05, **P < 0.01, ***P < 0.001, ****P < 0.0001 by two-tailed Student’s *t* test. Error bars indicate ± SEM.

Transcripts related to RNA splicing decreased in maternal *Nat10*-knockout 2-cell embryos, and we further used reverse transcription quantitative polymerase chain reaction (RT-qPCR) to detect transcript levels in WT and *Nat10*^*♀−/♂*^ 2-cell embryos. Consistent with the transcriptome sequencing results, levels of these genes were decreased in *Nat10*^*♀−/♂*^ 2-cell embryos (Fig. 4A). We performed mRNA stability assay that 50 μM α-amanitin was used to repress de novo gene transcription in cultured WT and *Nat10*^*♀−/♂*^ early 2-cell embryos. The mRNA levels of the examined transcripts gradually decreased over time in WT embryos with time, whereas the apparent decay kinetics of *Srsf2*, *Srsf4*, *Srsf9*, *Srsf10,* and *Zc3h14* were significantly accelerated in *Nat10*^*♀−/♂*^ 2-cell embryos (Fig. 4B).

Alternative splicing (AS) during mouse preimplantation development is an extremely complicated process, and stage-linked splicing is associated with embryonic development. SF3B1 and SRSF2 (SC35) are key splicing factors that localize to the nuclear speckles and mediate RNA splicing. Therefore, we examined whether the protein expression levels of nuclear speckle components, which are critical for RNA splicing, were altered in *Nat10*^*♀−/♂*^ 2-cell embryos. We first observed that SRSF2 and PABPN1 showed strong co-localization and formed numerous small speckles in WT growing oocytes. Quantitative analysis of immunofluorescent staining revealed that in *Nat10*^*oo–/–*^ oocytes, these speckles became significantly larger and fewer in number (Fig. 4C, D). Interestingly, during ZSA, SRSF2 reorganized into distinct speckle-like structures beginning at the 2-cell stage. However, in *Nat10*^*oo–/–*^ embryos, 47.83% of 2-cell embryos completely lacked nuclear speckles, 39.13% exhibited a marked reduction in speckle number, and only a minority formed normal speckles (Fig. 4E–G). To our knowledge, nuclear speckles are enriched with pre-mRNA processing factors, including splicing factors involved in AS, and ZC3H14 and p-T313-SF3B1 are important nuclear speckle markers. Immunofluorescence analysis showed reduced ZC3H14 and p-T313-SF3B1 signals in *Nat10*^*♀−/♂*^ 2-cell embryos, consistent with the above findings (Fig. 4F, G). Western blotting further confirmed decreased nuclear speckle marker expression upon maternal *Nat10* deletion (Fig. 4H). These findings demonstrated that NAT10 is important for the stability and expression of certain mRNA processing factors in nuclear speckles during ZSA.

### Global translation decreased in arrested two-cell embryos of maternal *Nat10* deletion

Considering that maternal deletion of *Nat10* in embryos impairs nuclear speckles protein synthesis and NAT10-dependent ac⁴C modification promotes translation, we first incubated *Nat10*^*♀−/♂*^ 2-cell embryos with L-homopropargylglycine (HPG), a methionine analog, to assess the global translational activity. The results showed a significant decrease in the translational activity of *Nat10*^*♀−/♂*^ 2-cell embryos (Fig. 4I, J).

Next, we conducted low-input ribosome profiling in conjunction with a ligation-free library preparation and sequencing (Ribo-lite) protocol using approximately 50 embryos at the 1-cell and 2-cell stages. Despite the slightly lower clean read rates, as expected from rRNA deletion skipping (Fig. S4G), Ribo-lite detects comparable numbers of expressed genes, and good correlations were observed in all samples (Fig. S4H). In *Nat10*^♀−/♂^ 2-cell embryos, ribosome profiling revealed downregulated ribosome protected fragments (RPFs) for 5027 transcripts and upregulated RPFs for 3766 others (Fig. S5A) These results indicate that maternal *Nat10* deletion is associated with widespread alterations in ribosome occupancy at the 2-cell stage (Fig. S5A, S7A–C). Interestingly, the RPFs enrichment levels of the ac^4^C^+^ gene transcripts were higher than those of the ac^4^C^-^ genes (Fig. S5B). We also overlapped the RPF translatome and the transcriptome of 2-cell embryos to assess the impacts of the global translatome at the transcript level during the ZGA transition, and crucially, we found that 431 ac^4^C^+^ gene transcripts that were markedly downregulated in *Nat10*^*♀−/♂*^ 2-cell embryos showed much lower RPF abundance in translatome (Fig. S5C, D). GO analysis of these 431 ac^4^C^+^ genes indicated that they were predominantly enriched in mRNA processing, transcriptional regulation, cell differentiation, and RNA splicing (Fig. S5E).

### NAT10 modulates zygotic splicing factors in an ac^4^C-dependent manner

**Fig. 5:**
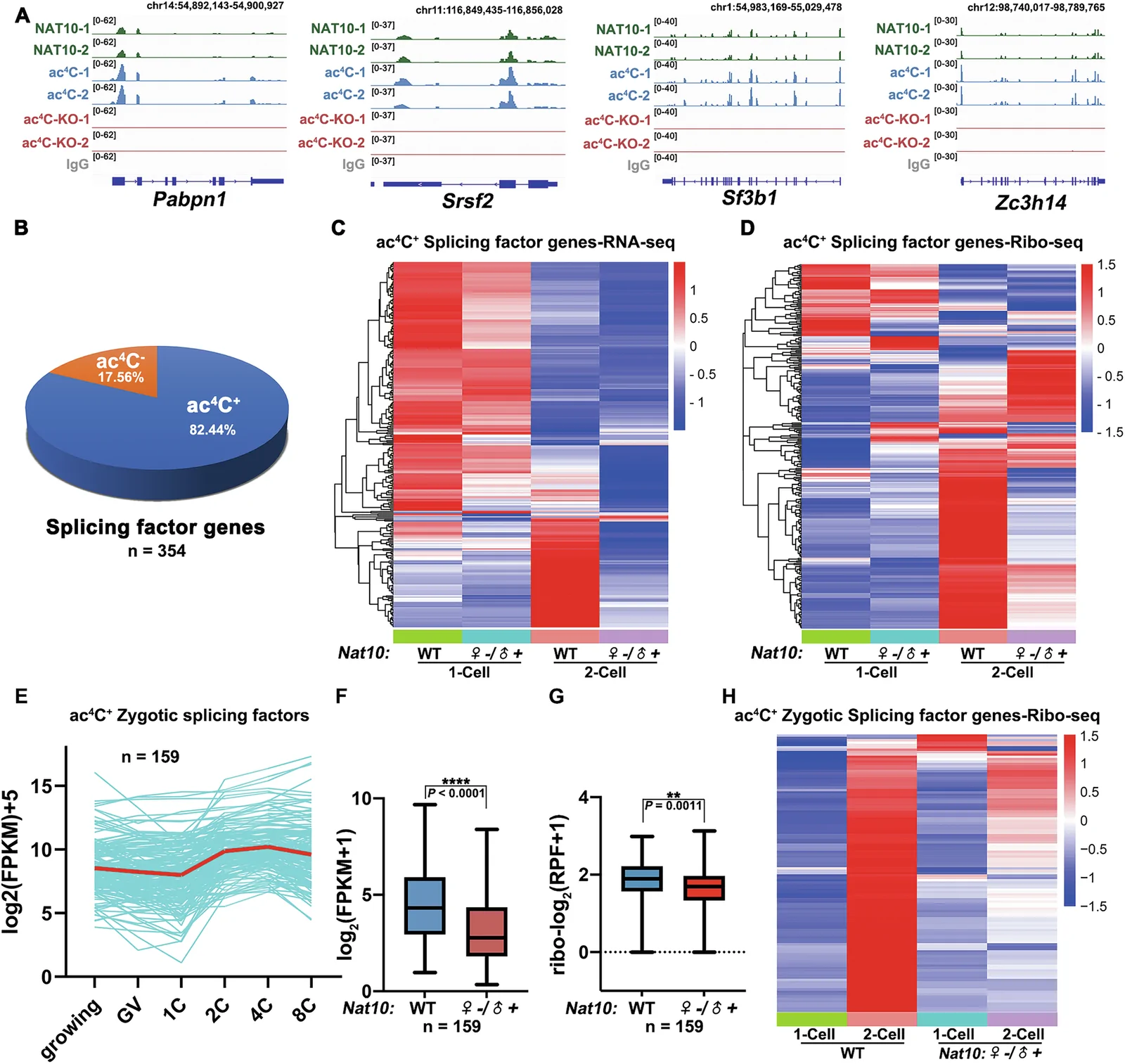
Maternal NAT10 supports zygotic splicing-factor transcripts linked to ac^4^C modification. **A** UCSC genome browser views of the reduced or lost ac^4^C peaks in key genes (*Pabpn1, Srsf2, Sf3b1* and *Zc3h14*) of WT and *Nat10*-null oocytes identified by LACE-seq. **B** Pie chart showing overlap between ac^4^C targets identified by LACE-seq and splicing factor genes. **C**, **D** Heatmap of fold enrichment scores for the characteristics of ac^4^C^+^ splicing factor genes at the mRNA (**C**) and RPF (**D**) levels in WT and maternal NAT10-deleted 2-cell embryos. **E** Line graphs showing the mRNA abundance of zygotic splicing factors in mouse oocytes and early preimplantation embryos at different stages. The gene number is 159. **F**, **G** Box plots showing the mRNA (**F**) and RPF (**G**) levels of zygotic splicing factors in 2-cell embryos of WT and *Nat10*^*fl/fl*^*;Zp3-Cre*. **H** Heatmap of ac^4^C^+^ zygotic splicing factor genes showing downregulated RPF expression in maternal NAT10-deleted 2-cell embryos. **P < 0.01, ****P < 0.0001 by two-tailed Student’s *t* test. Error bars indicate ± SEM.

Since the maternal deletion of *Nat10* in embryos affected mRNA processing and splicing, we wondered whether NAT10 regulates these target genes via ac^4^C modification. We then used Integrative Genomics View (IGV) software to search for the distribution of ac^4^C peaks on targets based on our ac^4^C LACE-seq datasets and found that the ac^4^C peaks associated with splicing were significantly decreased in *Nat10* knockout oocytes (Fig. 5A and Fig. S6A–F).

During the transition from oocytes to early embryonic stage, we selected a subset of splicing factor genes that play crucial roles based on available RNA-seq datasets. Notably, comparative analyses of NAT10-dependent ac^4^C-modified transcripts revealed 82.44% of splicing factor genes exhibit ac^4^C modification (Fig. 5B). Further analysis was conducted on these ac^4^C^+^ splicing factor genes, and it was found that these gene expression patterns were altered at both the mRNA abundance and ribosome-occupancy levels during the zygote to 2-cell transition, as evidenced by our RNA-Seq and Ribo-Seq datasets (Fig. 5C, D). By analyzing genes with significant changes in translational activity during MZT process, we divided these ac^4^C^+^ splicing factor genes into three major modes of translational dynamics: ‘ac^4^C^+^ zygotic splicing factors’ genes are upregulated after fertilization, particularly following ZGA; ‘ac^4^C^+^ maternal splicing factors’ and ‘ac^4^C^+^ stable splicing factors’ are downregulated and stable after fertilization, respectively (Fig. 5E, S7D, G, and Table S6). More genes belong to ac^4^C^+^ zygotic splicing factors, but their transcriptional and translational levels were significantly decreased in *Nat10*^*♀−/♂*^ 2-cell embryos (Fig. 5E–H). In comparison, fewer genes belonged to the maternal and stable splicing factors, with almost no effect on gene expression (Fig. S7D–I). These findings link NAT10 -dependent ac^4^C modification to the maintenance of zygotic splicing factors’ transcripts abundance and ribosome occupancy, providing a plausible mechanism for the alternative-splicing defects observed in maternal *Nat10*-deleted embryos.

### NAT10 regulates the alternative splicing of transcripts that function during zygotic splicing activation

**Fig. 6:**
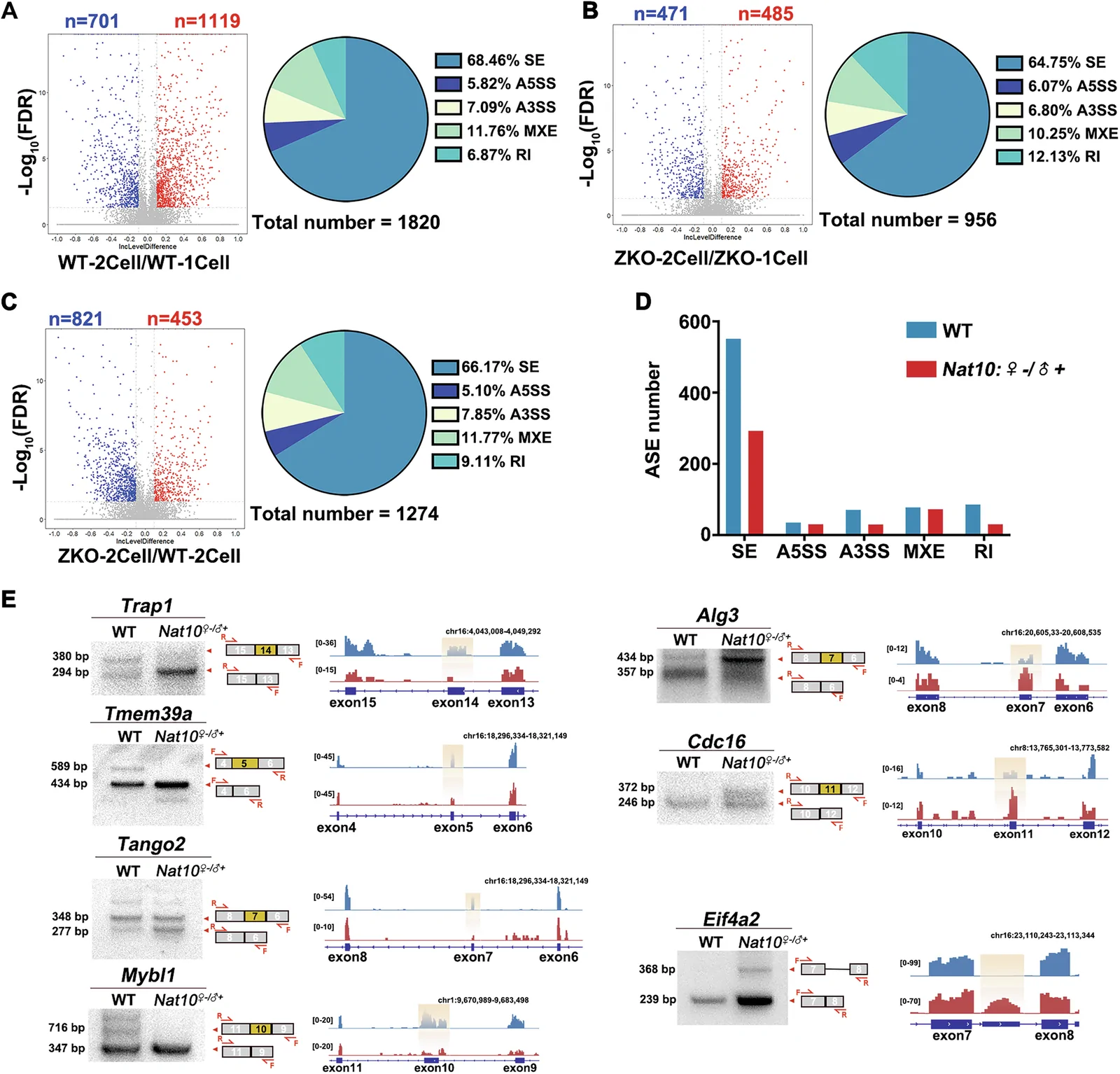
Maternal NAT10 deletion impairs alternative splicing during MZT. **A** Alternative splicing events (ASEs) at different stages in WT 1-cell and 2-cell embryos are shown with a volcano map, and the ratio of different ASEs in WT mouse embryos at the 2-cell stage. **B** ASEs at different stages in maternal *Nat10*-knockout 1-cell and 2- cell embryos are shown with a volcano map, and the ratio of different ASEs in maternal *Nat10*-knockout embryos at the 2-cell stage. **C** Volcano plot showing changes in transcript isoforms skipped or included in 2-cell embryos derived from WT and *Nat10*^*fl/fl*^*;Zp3-Cre* females mated with WT males. Five types of ASEs were identified in maternal *Nat10*-knockout embryos at the 2-cell stage. **D** Distribution of five ASEs in 2-cell embryos of WT and *Nat10*^*fl/fl*^*;Zp3-Cre* females mated with WT males. SE, skipped exon, MXE, mutually exclusive exon, RI, retained intron, A5SS, alternative 5’ splice site, A3SS, alternative 3’ splice site. **E** Validation of different transcript isoforms of genes in 2-cell embryos of WT and *Nat10*^*fl/fl*^*;Zp3-Cre* females mated with WT males by reverse transcription-polymerase chain reaction (RT-PCR) and visualization results from the Integrative Genomics Viewer.

As ZSA occurs during ZGA, to further explore the role of maternal *Nat10* in early embryonic development, we analyzed alternative splicing events (ASEs) using RNA-Seq data. A large number of ASEs occur in the zygote to 2-cell stage of both WT and *Nat10*^*♀−/♂+*^ embryos, and there was no significant difference in the proportion of ASEs (Fig. 6A, B). However, the number of detectable ASEs after maternal *Nat10* knockout in 2-cell decreased, and categorical analysis of these events revealed a predominant decrease in all common ASE categories (Fig. 6C, D). Several ASEs were selected to validate the findings.

RT-qPCR analysis of WT and *Nat10*^*♀−/♂+*^ 2-cell embryos using primers flanking the skipping exons of target genes: *Trap1*, *Tmem39a*, *Tango2* and *Mybl1*, and verified that the exon inclusion levels of these four genes were all significantly decreased in the *Nat10*^*♀−/♂+*^ embryos (Fig. 6E). In contrast, *Alg3* and *Cdc16* did not undergo alternative splicing at the 2-cell stage, but skipped exon 7 and 11 after maternal *Nat10* knockout, respectively (Fig. 6E). Another gene, Eif4a2, which is associated with translation initiation factor activity, showed splicing of the intron between exon 7 and 8 at the 2-cell stage, whereas IR occurred after maternal *Nat10* knockout (Fig. 6E). Together, these analyses indicate that maternal *Nat10* deletion is associated with widespread disruption of the zygotic alternative-splicing program in 2-cell embryos.

### Zygotically overexpression of NAT10 failed to rescue the developmental defects of maternal NAT10-deficient embryos

**Fig. 7:**
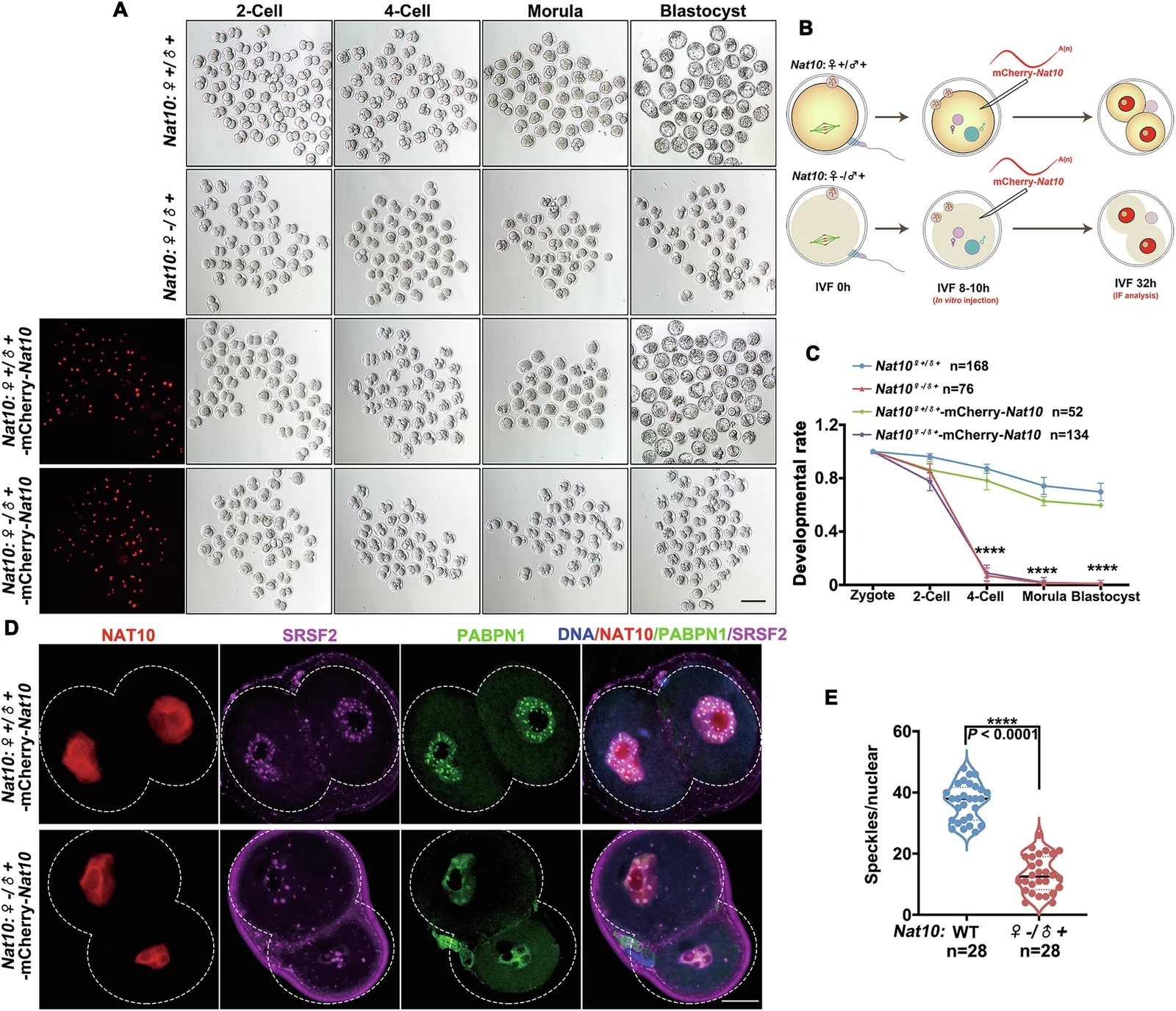
Supplementing NAT10 in maternal NAT10-deficient zygotes failed to rescue the developmental defects and the assembly of nuclear speckles. **A** Representative image of mCherry-*Nat10* mRNA microinjected embryos and non-injected controls at different developmental stages. Fluorescence images indicate the expression efficiency of the in vitro transcribed mRNA. Scale bar = 100 μm. **B** Schematic diagram showing the microinjection time, sample collection for immunofluorescence, and their impact on early embryonic development (**A**). **C** Zygotes collected by in vitro fertilization were microinjected with mCherry-*Nat10* mRNA and cultured for 96 h. The development rates of embryos at the indicated stages were evaluated. The number of embryos analyzed is shown (n). Error bars, S.E.M. ***P < 0.001 by two-tailed Student’s *t* test. ns: non-significant. **D** Immunofluorescence images indicating the status of nuclear speckles in 2-cell after injection of NAT10 into the zygote. Scale bar = 20 μm. **E** Quantification of nuclear speckles in 2-cell embryos. The number of nuclei analyzed is indicated (n). Error bars indicate ± SEM. ****P < 0.0001 by two-tailed Student’s *t* test.

To further confirm the physiological role of NAT10 during preimplantation embryonic development, mRNA (800 ng/μL) encoding NAT10 and mCherry was injected into the cytoplasm of *Nat10*^*♀+/♂+*^ and *Nat10*^*♀–/♂+*^ zygotes at IVF 8–10 h to investigate the effect of NAT10 supplementation on embryos in vitro development (Fig. 7B). Detection of the exogenous NAT10 protein and ac^4^C modification confirmed that the *mCherry-tagged Nat10* mRNA used in this study was full-length, fully functional, present in the cells, and biologically active (Fig. S6G–I). The mCherry fluorescence in 2-cell embryos confirmed expression of the injected mCherry-*Nat10* mRNA, and exogenous NAT10 was detected in both the nucleolus and nucleoplasm (Fig. 7A, D). Consistent with what we observed for the *Nat10*^*♀+/♂+*^ and *Nat10*^*♀–/♂+*^ embryos, the development of *Nat10*^*♀–/♂+*^ embryos microinjected with *Nat10* mRNA was arrested at 2-cell stage, while the *Nat10*^*♀+/♂+*^ embryos microinjected with *Nat10* mRNA successfully developed into blastocysts similar to the wild type control group (Fig. 7A–C). This indicated that maternal NAT10 plays more critical functions for early embryonic development from the 2-cell to 4-cell stage than zygotic, and even exogenous NAT10 overexpression in *Nat10*^*♀–/♂+*^ embryos failed to effectively rescue embryonic developmental rates. The 2-cell stage embryos were collected and monitored for potential NS assembly at 32 h after IVF, and SRSF2 and PABPN1 were selected as nuclear speckle markers to examine speckle localization and expression levels. In *Nat10*^*♀–/♂+*^ 2-cell embryos microinjected with *Nat10* mRNA, virtually all embryos were capable of forming nuclear speckles (Fig. 7D, E). However, the number of speckles decreased, but the size of the speckles increased compared to the control (Fig. 7D, E). These findings indicate that zygotic overexpression of NAT10 partially rescued nuclear speckle assembly defects in maternal NAT10-deficient embryos. Thus, zygotic NAT10 overexpression can partially restore nuclear-speckle-like structures but does not fully normalize speckle architecture or developmental competence.

### NAT10 acetylates and interact with SRSF2 to modulate the nuclear speckle

NAT10 also participates in various cellular and biological processes by exerting acetyltransferase activity on target proteins to regulate protein stability. Recent studies revealed that NAT10 interacts with the splicing factor SRSF2, leading to its acetylation and increased stability in gastric cancer, ultimately regulating splicing events [38]. Because acetylation can influence the stability, localization and activity of splicing-related proteins, we asked whether NAT10-dependent regulation of SRSF2 might contribute to nuclear speckle organization in oocytes and early embryos.

**Fig. 8:**
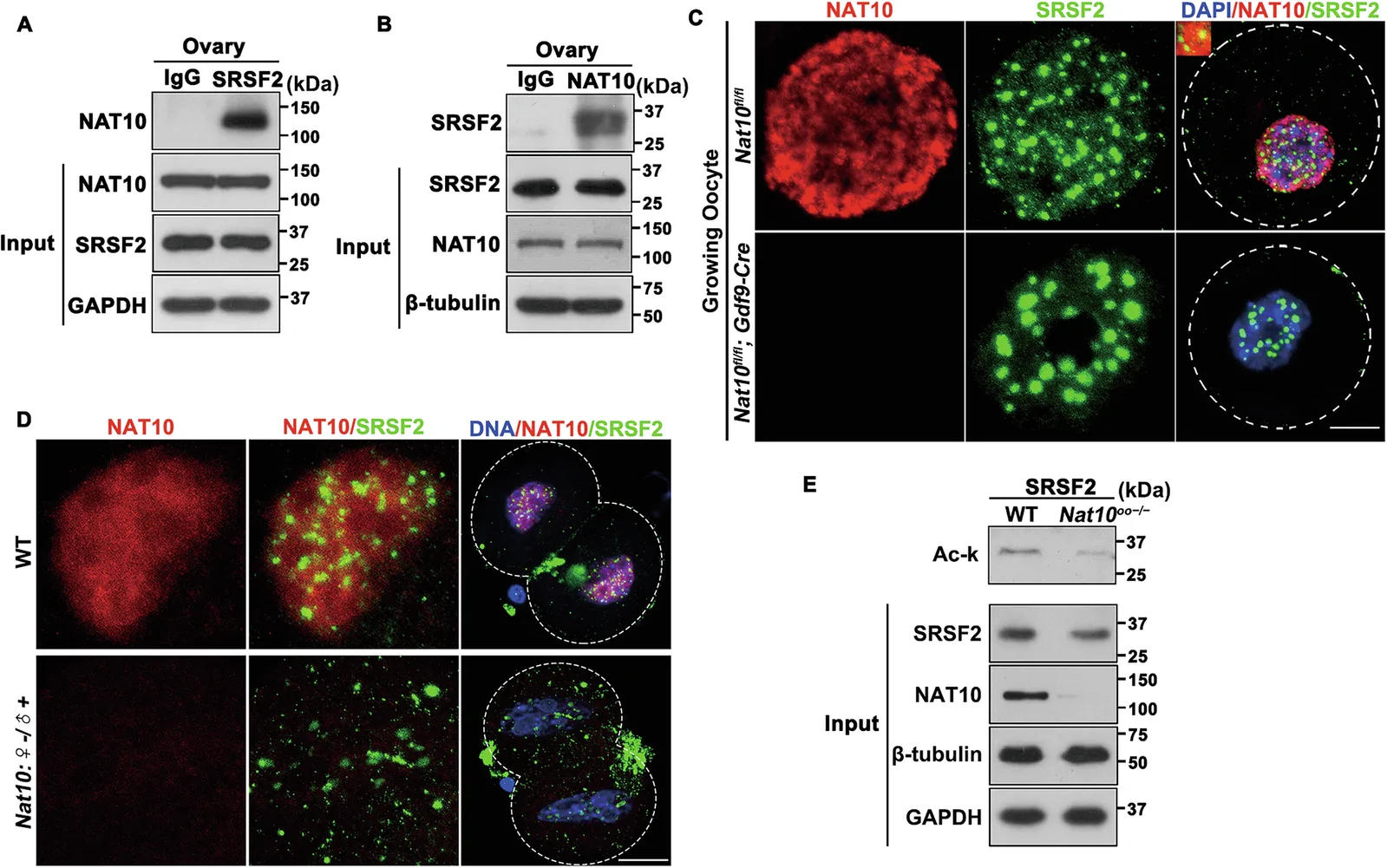
NAT10 is essential for the reconstitution and acetylation of SRSF2. **A**, **B** Co-IP analyses were performed in mouse ovaries using anti-NAT10 (**A**) or anti-SRSF2 (**B**) antibodies, or IgG as a control. Endogenous SRSF2 (**A**) and NAT10 (**B**) in the resulting immunoprecipitates were detected by western blotting. **C**, **D** Confocal image showing the co-localization of NAT10 and SRSF2 fusion staining in growing oocytes (**C**) and 2-cell embryos (**D**) of WT and *Nat10*^*fl/fl*^*;Gdf9-Cre* female mice. Scale bar, 20 μm. **E** Co-immunoprecipitation experiments were performed using an anti-SRSF2 antibody in growing oocytes of WT and *Nat10*^*fl/fl*^*;Zp3-Cre* female mice.

Therefore, we examined whether maternal *Nat10* knockout–induced embryonic arrest was associated with defects in these processes. Immunoprecipitation (IP) assays using mouse ovarian tissues showed reciprocal interaction between NAT10 and SRSF2 (Fig. 8A, B). Immunofluorescence further demonstrated NAT10–SRSF2 colocalization in growing oocytes and 2-cell embryos. Upon maternal *Nat10* knockout, NAT10 signals were nearly undetectable, SRSF2 expression and localization were disrupted, and nuclear speckle organization was impaired, with enlarged but fewer speckles in oocytes and markedly reduced speckle numbers in *Nat10*^*♀−/♂*^ 2-cell embryos (Fig. 8C, D). These results supported the hypothesis that maternal NAT10 deficiency disrupts SRSF2 expression and subcellular localization. Additionally, IP experiments by using SRSF2 antibody in WT and *Nat10* knockout growing oocytes revealed that *Nat10* deletion significantly reduces acetylation levels on SRSF2, which suggested that these regulatory effects and even the mechanism of *Nat10*^*♀−/♂*^ 2-cell arrest were results of SRSF2 acetylation by NAT10. These findings support a model in which NAT10 functionally interacts with SRSF2 and may regulate nuclear speckle organization and alternative splicing partly through SRSF2 acetylation. The precise molecular crosstalk between NAT10-mediated RNA acetylation and SRSF2 protein acetylation remains to be further defined.

## Discussion

NAT10 is the currently established mammalian writer of RNA ac^4^C and has been implicated in diverse physiological processes and contribute to progression of disease [11, 12, 25, 44–46], and an increasing number of studies have shown that serial epigenetic events play diverse roles in regulating the composition of oogenesis-associated maternal mRNAs [19, 24, 33, 43, 47]. For instance, emerging evidence has revealed the essential roles of m^6^A writers and readers in oogenesis and early embryogenesis [19, 47–50]. While m⁶A is a highly prevalent mRNA modification, the presence and functional role of ac⁴C in mammalian mRNA remain controversial [51–53]. The involvement of NAT10-mediated ac^4^C in mouse embryonic development has been reported [43, 54]. However, the maternal contribution of NAT10 to early embryonic development and its relationship with post-transcriptional regulation during the maternal-to-zygotic transition remain incompletely understood. Here, we demonstrate the detrimental effect of maternal *Nat10* deletion at the two- to four-cell stages and identify critical NAT10-mediated ac^4^C target genes on splicing factors that alter the expression of these genes, which perturbs alternative splicing patterns, leading to aberrant isoform switching in cellular physiology. As the ac^4^C writer, NAT10 and ac^4^C exhibit dynamic expression, especially at the two-cell stage, and play key roles during preimplantation development. Different from the phenotypes of *Nat10* knockout using *Gdf9-Cre* as early as the primordial follicle stage [32], *Nat10* deletion in oocytes from primary follicles has little effect on oocyte meiotic maturation; however, maternal *Nat10* knockout severely impairs preimplantation development, with most embryos arresting at the 2-cell stge. Our results indicate that the downregulation of ZGA transcripts is dramatic in maternal NAT10-deficient 2-cell embryos accompanied by an abnormal accumulation of maternal transcripts. This phenomenon indicates that NAT10’s function in regulating coding genes during the MZT depends on the direct recognition of ac^4^C. We further found that NAT10 affected the stability and expression of nuclear speckles, and furthermore, NAT10 modulates these zygotic splicing factors in an ac^4^C-dependent manner. Finally, drastic reprogramming of ac^4^C-dependent transcripts upon fertilization disrupts pre-established alternative splicing patterns in maternal *Nat10*-deficient 2-cell embryos during zygotic splicing activation.

The effect of ac^4^C modification on mouse preimplantation embryonic development has been reported to influence the transition from the morula to the blastocyst stage and the first cell fate decision in mice [43]. Mechanistically, *Nat10* knockdown by injecting siRNA against *Nat10* decreases the ac^4^C modification of *Nop2* mRNA and reduces RNA and protein abundance, resulting in defective expression of the downstream lineage-specific gene *Cdx2*, ultimately preventing blastomeres from undergoing trophectoderm fate [43]. However, the siRNA injection method cannot deplete maternal proteins in zygotes and 2-cell embryos, and the underlying functions of maternal NAT10 in early embryonic development remain unclear. Furthermore, our results demonstrated that the loss of maternal *Nat10* results in the characteristic phenotypes of developmental arrest at the 2-cell stage, including abnormal accumulation of maternal transcripts and down-regulation of ZGA inactivation. Importantly, our data on splicing factors and nuclear speckles highlight the post-transcriptional and translational regulation that occurs through NAT10-mediated ac^4^C modification, which may also play important roles in multiple developmental events. Our RNA-seq and Ribo-lite data of wild-type control and NAT10-deficient 2-cell embryos revealed the dysregulation of zygotic splicing factor transcripts, including *Srsf2*, *Pabpn1*, *Sf3b1* and *Zc3h14*, all of which are crucial for the alternative splicing process. The maternal NAT10-deficient embryos show disrupted splicing factor reactivation and reorganization during early embryonic development, especially at the 2-cell stage. We analyzed alternative splicing alterations and discovered that all categories of ASEs decreased. These findings indicated that the disruption of splicing factor expression due to M-decay or the failure of ZGA, based on decreased ac^4^C modification during MZT, resulted in the failure of ZSA.

Our study not only elucidates the essential regulatory function of NAT10 in post-transcriptional regulation through ac⁴C modification of RNA, but also provides definitive evidence that NAT10 directly modulates the activity of nuclear speckle-associated proteins via protein acetylation pathways, thereby compromising the assembly and physiological functionality of nuclear speckles. Beyond its role in post-transcriptional regulation, NAT10 directly interacts with the nuclear speckle-associated splicing factor SRSF2 and promotes its acetylation, thereby maintaining SRSF2 stability and nuclear speckle integrity. Maternal NAT10 deficiency reduces SRSF2 acetylation, disrupts nuclear speckle organization, and compromises splicing fidelity, leading to aberrant alternative splicing and developmental arrest. Alternative splicing expands transcriptomic diversity and regulatory capacity through post-transcriptional RNA processing [38, 55, 56]. In oocytes, maternal splicing factors are essential for meiotic progression, oocyte maturation, and female fertility [41, 42, 57, 58]. During early embryogenesis, alternative splicing further shapes transcriptional dynamics and is activated after embryonic genome activation as part of zygotic splicing activation [36]. Notably, this process begins at the 2-cell stage in mouse preimplantation embryos, coinciding with the totipotent-to-pluripotent transition [59–61]. This epitranscriptomic reprogramming mechanistically governs embryonic stem cell potency conversion, as established in multiple studies [60, 62]. Our work underscores an additional role of NAT10-mediated ac^4^C for modulating gene expression of splicing factors and indicates that ac^4^C modification is not a mere side effect of epigenetic reprogramming but instead plays an important role in ZSA of the early embryonic development in mice and examined the mechanism underlying embryonic developmental arrest resulting from disruptions in alternative splicing.

In mouse oocytes and preimplantation embryo development, ac^4^C modification is believed to play a pivotal role; however, we lack detailed knowledge of the molecular mechanisms involved in the application of low-input detection technologies. In our previous study, we developed an ac^4^C LACE-seq with high sensitivity and low input compared to other published ac^4^C detection technologies and revealed 8,241 ac^4^C peaks in GV-stage oocytes, which were reduced or eliminated after the deletion of *Nat10*. A limitation of this study is that we have not directly mapped the ac^4^C landscape at the 2-cell stage, the key developmental window affected by maternal *Nat10* deletion. This limits our ability to distinguish ac^4^C marks inherited from oocytes from those newly deposited after fertilization or on newly synthesized zygotic transcripts. Because 2-cell embryos provide extremely limited input material and undergo rapid RNA remodeling during the maternal-to-zygotic transition, robust ac^4^C profiling at this stage remains technically challenging. Thus, our ac^4^C LACE-seq data primarily define NAT10-dependent ac^4^C deposition in oocytes, and the ac^4^C status of splicing-factor transcripts in 2-cell embryos may partly reflect modifications established during oocyte growth. Nevertheless, the genetic arrest phenotype, abnormal maternal transcript persistence, attenuated ZGA transcript accumulation, reduced ribosome occupancy of splicing-related transcripts, impaired nuclear speckle organization and altered alternative-splicing patterns collectively support a model in which maternal NAT10 coordinates ac^4^C -associated post-transcriptional regulation during zygotic splicing activation. Future high-sensitivity ac^4^C mapping in low-input embryos will be needed to directly resolve inherited versus newly deposited ac^4^C marks during major ZGA.

## Supplementary information

Figure S1

Figure S2

Figure S3

Figure S4

Figure S5

Figure S6

Figure S7

Supplemental Figure Legend

Supplementary Table 1-6

Original WB

## Data Availability

RNA-seq and Ribo-lite sequencing raw data have been deposited in the NCBI Gene Expression Omnibus database (https://www.ncbi.nlm.nih.gov). The RNA-seq raw data is under accession code with GSE308580. The Ribo-lite sequencing data created in this study are available from the GEO database, and the accession number is GSE308284. The previously published ac^4^C LACE-seq data used in this work was from NCBI GEO accession number GSE253976. The data required to corroborate the conclusions of this work are contained within this manuscript as well as the accompanying Supplementary Materials.
