# Supplementary material for: The RNA ac4C writer NAT10-dependent post-transcriptional regulations are prerequisites for zygotic splicing activation in mouse
Source: Cell Death Dis. 2026 Aug 17;17(1):962. doi: 10.1038/s41419-026-09186-y (PMC13754847; doi:10.1038/s41419-026-09186-y)
Supplement: Supplementary file 1 [file 41419_2026_9186_MOESM8_ESM.docx]

**Supplemental Figure Legend**

**Figure S1. High-efficiency *Nat10* knockout from mouse primary follicle oocytes.**

**A** Quantification of the western blot result in Figure 1B. n = 2.**B** Relative NAT10 fluorescence intensity result in Figure 1C. n = 4. **C** Relative ac^4^C fluorescence intensity result in Figure 1D. n = 4. **D** Reanalysis of published data on *NAT10* mRNA expression in human oocytes and early pre-implantation embryos. **E** Immunohistochemistry showing NAT10 protein levels in the ovaries of 4-week-old WT and *Nat10^fl/fl^;Zp3-Cre* mice. Follicle stages are shown as indicated. Arrows and hollow arrows indicate primordial follicles and oocyte nuclei of growing follicles, respectively. **F** Immunofluorescence was used to detect the expression and localization of NAT10 and Lamin B1 proteins in GV-stage oocytes of WT and *Nat10^fl/fl^;Zp3-Cre* mice.

**Figure S2. Validation of oocyte-specific Nat10 deletion and assessment of ovulation and meiotic maturation. A** Representative images showing the morphology of the ovaries of 4-week-old mice with the indicated genotypes before and after PMSG injection for 44 h. Scale bar, 500 μm **B** Hematoxylin and eosin-stained images showing the histology of 4-week-old WT and *Nat10^fl/fl^;Zp3-Cre* ovaries. Scale bar, 100 μm. **C** Comparison of ovary weights between 4-week-old WT and *Nat10^fl/fl^;Zp3-Cre* female mice. **D** Ovary weight of 4-week-old WT and *Nat10^fl/fl^;Zp3-Cre* female mice after hCG injection for 20 h. **E** Number of oocytes ovulated by 4-week-old mice with the indicated genotypes after superovulation. Data was expressed mean ± SEM. **F** Ratio of SN-type oocytes isolated from 4-week-old *Nat10^fl/fl^* and *Nat10^fl/fl^;Zp3-Cre* mice. Data was presented as mean ± SEM. **G** PB1 emission rates of oocytes in vivo collected at the MII stage from the oviducts of WT and *Nat10^fl/fl^;Zp3-Cre* mice 16 h after hCG injection. The total number of oocytes analyzed (n) is shown. ns, non-significant according to two-tailed Student’s t-test. Error bars indicate ± SEM. **H** Immunofluorescence staining of Ac α-tubulin showing spindles assembly in WT and *Nat10^fl/fl^;Zp3-Cre* oocytes after culture for 16 h in vivo. The total number of oocytes used is indicated in the corners (oocytes showing the percentage of representative phenotypes and total oocytes observed). **I** Western blot analysis showing expression levels of the indicated proteins in germinal vesicle (GV) and metaphase MII (metaphase II) oocytes collected from WT and *Nat10^fl/fl^;Zp3-Cre* mice. DDB1was used as the loading control. The total protein from 100 oocytes was loaded into each lane. **J** Immunofluorescence of Nanog and CDX2 in the WT and *Nat10^fl/fl^;Zp3-Cre* blastocyst stages. ICM, inner cell mass; TE, trophectoderm. Scale bar, 20 μm. **K** Quantification of blastomere number in blastocysts from each experimental group. n.s.: not significant; *P < 0.05, ***P < 0.001 by two-tailed Student’s t-test. Error bars indicate ± SEM.

**Figure S3. Phenotype Characterization of Follicle Development and Ovulation. A** Immunofluorescence results showing the expression and localization of ac^4^C in WT and *Nat10^fl/fl^;Zp3-Cre* oocytes, which contained both non-surrounded nucleolar (NSN) and surrounded nucleolar (SN) chromatin configurations. More than 30 oocytes of each type were observed with similar results. **B** Quantitative statistics of the ac^4^C fluorescence signal in (A). **C** Cumulus-oocyte complex and denuded GV stage oocytes derived from 4-week-old *Nat10^fl/fl^* and *Nat10^fl/fl^;Zp3-Cre* mice. **D** Rates of germinal vesicle breakdown (GVBD) and polar body-1 (PB1) emission in oocytes cultured in vitro. Fully grown oocytes were collected from PMSG-treated (44 h) mice of the indicated genotypes. The number of oocytes analyzed is shown (n). **E** Quantification of Ac α-tubulin signal intensity in MII oocytes in each experimental group. **F** Hematoxylin and eosin-stained images showing histological changes during hCG-induced ovulation in WT and *Nat10^fl/fl^;Zp3-Cre* ovaries at 20 h post-hCG injection. **G** Confocal microscopic images of immunofluorescence for α-tubulin showing spindles assembly in WT and *Nat10^fl/fl^;Zp3-Cre* oocytes after culture for 16 h in vitro. The total number of oocytes used is indicated in the corners (oocytes showing the percentage of representative phenotypes and total oocytes observed). Scale bar, 20 μm. *P < 0.05, ***P < 0.001 by two-tailed Student’s t-test. Error bars indicate ± SEM.

**Figure S4. Transcriptomics and translatomics analysis of oocytes and embryos from WT and *Nat10^fl/fl^;Zp3-Cre* female mice. A** Heat map of the Spearman correlation coefficients among WT and *Nat10^fl/fl^;Zp3-Cre* oocytes and embryos at the indicated developmental stages. **B-C** Scatter plots comparing the gene expression levels of WT and *Nat10^fl/fl^;Zp3-Cre* embryos at GV (B) and MII (C) stage. Three RNA-seq replicates were generated for differential gene expression analyses. **D** Venn diagrams showing the overlap in transcripts that were significantly decreased during GV to MII transition (fold change (GV/MII)>=4) of WT oocytes (M-Decay) and genes abnormally upregulated at 2-cell stage after *Nat10* maternal knockout.**E** Venn diagrams showing the overlap of transcripts that were significantly decreased in WT 2-cell and abnormally decreased in *Nat10^oo-/-^* GV oocytes. **F** Heatmap of fold enrichment scores for the characteristic of genes abnormally upregulated at 2-cell stage after *Nat10* maternal knockout on mRNA levels at the GV and MII stage between WT and *Nat10^fl/fl^;Zp3-Cre* mice. Each rectangle represents a gene category. **G** Bar plots showing the percentage of reads that are adapter, rRNA or clean reads (non-rRNA mapped reads) in different dataset. Results are from two independent experiments. **H** Heat map of the Spearman correlation coefficients among WT and *Nat10^fl/fl^;Zp3-Cre* embryos at the indicated developmental stages.

**Figure S5. Translatome analyses in maternal *Nat10*-knockout embryos. A** Scatter plot comparing the normalized ribosome protected fragment (RPF) abundance calculated via average FPKM between WT and maternal NAT10-deleted 2-cell embryos. RPFs that decreased or increased by more than 2-fold in maternal NAT10-deleted 2-cell embryos are highlighted in blue and red, respectively. **B** Box plots showing the relative RPF levels of genes with or without ac^4^C modification in WT and maternal NAT10-deleted 2-cell embryos. **C** Scatter plot comparing the translation efficiency (TE, RPF/mRNA) values between WT and maternal NAT10-deleted 2-cell embryos. **D** Scatter plot comparing gene transcript overlap between RPF fold change (log_2_ ratio) and mRNA fold change (log_2_ ratio) in WT and maternal NAT10-deleted 2-cell embryos. **E** Enriched functional terms for Gene Ontology biological processes (GO-BP) of the downregulated transcripts at both the mRNA and RPF levels (D).

**Figure S6. A-F** UCSC genome browser views of abolished ac^4^C peaks on the key genes (*Srsf1, Srsf4, Srsf5, Srsf9, Srsf10* and *Srsf11*) of WT and *Nat10*-null oocytes identified by LACE-seq. **G** Western blots indicating the expression level of mCherry-*Nat10* in vitro. Total proteins from 80 2-cells were loaded in each lane. GAPDH served as the loading control. **H** Immunofluorescence results showing the expression and localization of ac^4^C in the indicated genotype 2-Cell. The number of analyzed embryos in indicated (n). **I** Quantitative statistics of the ac^4^C fluorescence signal in (H). *P < 0.05 by two-tailed Student’s t-test. Error bars indicate ± SEM.

**Figure S7. NAT10 has less effect on maternal and stable splicing factors. A** Scatter plot comparing the ribosome protected fragment (RPF) values calculated via average FPKM between WT and maternal NAT10-deleted 1-cell embryos. RPFs that decreased or increased by more than 2-fold in maternal NAT10-deleted 1-cell embryos are highlighted in blue and red, respectively. **B** Scatter plot comparing the translation efficiency (TE, RPF/mRNA) values between WT and maternal NAT10-deleted 1-cell embryos. **C** Box plots showing relative TE values at the 1-cell and 2-cell stages. The box indicates the upper and lower quantiles; the thick line in the box indicates the median; and the whiskers indicate the 2.5th and 97.5th percentiles. **D** Line graphs showing the RPF dynamics of maternal splicing factors in mouse oocytes and early embryos at different stages. **E-F** Box plots showing the mRNA (E) and RPF (F) levels of maternal splicing factors in 2-cell embryos of WT and *Nat10^fl/fl^;Zp3-Cre.* **G** Line graphs showing the RPF dynamics of stable splicing factors in mouse oocytes and early embryos at different stages. **H-I** Box plots showing mRNA (H) and RPF (I) levels of stable splicing factors in 2-cell embryos of WT and *Nat10^fl/fl^;Zp3-Cre.* n.s.: not significant; *P < 0.05, ***P < 0.001 by two-tailed Student’s t-test. Error bars indicate ± SEM.
